# The Effects of Thiazolidinediones on Metabolic Complications and Lipodystrophy in HIV-Infected Patients

**DOI:** 10.1155/2009/373524

**Published:** 2008-12-01

**Authors:** Jussi Sutinen

**Affiliations:** Division of Infectious Diseases and Division of Diabetes, Department of Medicine, Helsinki University Central Hospital, 00029 HUS, Helsinki, Finland

## Abstract

Highly active antiretroviral therapy (HAART)-associated metabolic complications include lipoatrophy (loss of subcutaneous adipose tissue (SAT)) and insulin resistance. Thiazolidinediones are insulin-sensitizing antidiabetic agents which—as an untoward side effect in obese diabetic patients—increase SAT. Furthermore, troglitazone has improved lipoatrophy and glycemic control in non-HIV patients with various forms of lipodystrophy. These data have led to 14 clinical trials to examine whether thiazolidinediones could be useful in the treatment of HAART-associated metabolic complications. The results of these studies indicate very modest, if any, effect on lipoatrophic SAT, probably due to ongoing HAART negating the beneficial effect. The benefit might be more prominent in patients not taking thymidine analoges. Despite the poor effect on lipoatrophy, thiazolidin-ediones improved insulin sensitivity. However, especially rosiglitazone induced harmful effects on blood lipids. Current data do not provide evidence for the use of thiazolidinediones in the treatment of HAART-associated lipoatrophy, but treatment of lipoatrophy-associated diabetes may be warranted. The role of thiazolidinediones for novel indications, such as hepatosteatosis, should be studied in these patients.

## 1. Introduction

The prognosis of HIV-infection has drastically changed after the introduction of combination antiretroviral therapy [1] often referred to as highly active antiretroviral therapy (HAART). Since the eradication of the virus is impossible with current medicines [2] and since periodic treatment with HAART can be harmful when compared to continuous therapy [3], patients need to continue therapy uninterruptedly and permanently.

**Figure 1:**
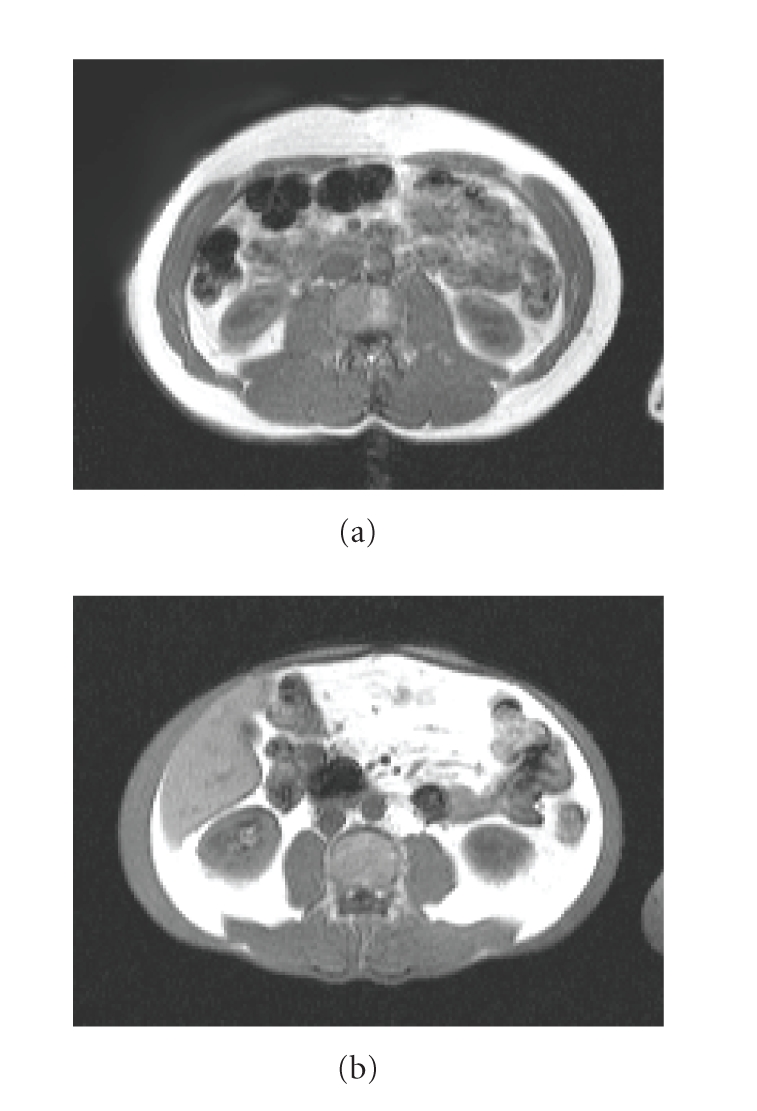
Abdominal magnetic resonance image (MRI) of a HAART-treated patient with normal fat distribution (a) and a patient with severe HAART-associated lipodystrophy with complete loss of subcutaneous fat and accumulation of intra-abdominal fat (b). Fat is shown white in these MRI images.

Lifelong exposure to HAART puts patients at a significant risk for long-term metabolic adverse effects including lipodystrophy, insulin resistance, hyperlipidemia, and increased cardiovascular morbidity [4, 5]. The most characteristic component of HAART-associated lipodystrophy is the loss of subcutaneous adipose tissue [6] which has proven to be very difficult to treat (Figure 1).

Thiazolidinediones (glitazones) are oral insulin-sensitizing antidiabetic agents. As an untoward side effect, glitazones increase subcutaneous fat mass in patients with type 2 diabetes [7–9]. Additionally, in non-HIV infected patients with various forms of lipodystrophy, troglitazone has improved metabolic control and subcutaneous lipoatrophy [10]. These insulin-sensitizing and fat-inducing effects of glitazones have lead to several clinical trials examining whether these drugs could reverse lipoatrophy and insulin resistance in patients with HAART-associated lipodystrophy.

The ensuing review is focused on summarizing the currently available clinical data on the use of glitazones in patients with HAART-associated lipodystrophy

## 2. Thiazolidinediones

Thiazolidinediones are synthetic ligands for peroxisome proliferator-activated receptor gamma (PPAR*γ*). PPAR*γ* is a nuclear receptor which alters expression of multiple genes including those regulating lipid and glucose metabolism [11]. PPAR*γ* is expressed mainly in adipose tissue and is also found in pancreatic beta cells, vascular endothelium, and macrophages, and in low quantities in other tissues such as the liver, skeletal muscle, and the heart [12, 13]. The activation of PPAR*γ* is critical in adipocyte differentiation, fatty acid uptake, and storage in the adipocytes [14, 15].

Glitazone-induced activation of PPAR*γ* in adipose tissue may improve the whole body insulin sensitivity by keeping fatty acids within adipocytes and hence protecting other tissues (liver, skeletal muscle, and pancreatic beta cells) from the “toxic” effects of the high-circulating concentration of free fatty acids [12]. Alternatively or additionally, glitazones may affect whole body insulin sensitivity by altering adipokine release from adipose tissue [12].

The potential role of PPAR*γ* in the pathogenesis of some human lipodystrophies has been demonstrated in recent studies finding dominant negative and loss-of-function mutations to affect the ligand-binding domain of PPAR*γ* in non-HIV patients with partial lipodystrophy, hepatic steatosis, dyslipidemia, and insulin resistance [13]. Furthermore, trogilitazone was shown to improve subcutaneous lipoatrophy in HIV-negative patients with various forms of lipodystrophic/lipoatrophic syndromes [10]. Taken together, the available data make glitazones an interesting therapy option for HAART-associated lipodystrophy.

## 3. HAART-Associated Lipodystrophy

The prevalence of HAART-associated lipodystrophy has varied from as low as 2% [16] up to 83% [17] in HAART-treated patients. This huge variation is explained by the lack of uniformly accepted definition of lipodystrophy, and the variable combination and duration of HAART in different studies. Estimates from large surveys indicate a prevalence of 50% of at least one physical abnormality after 12–18 months of therapy [18, 19]. Most of these prevalence data arise from patients taking mainly the older and metabolically more toxic antiretroviral regimens. Although there are accumulating data demonstrating a significantly decreased risk for lipodystrophy in patients taking newer antiretroviral agents [20, 21], lipodystrophy still remains a significant clinical problem.

Lipoatrophy, that is, a decrease in subcutaneous adipose tissue (SAT) mass, has mainly been attributed to the use of nucleoside reverse transcriptase inhibitors (NRTIs) and thymidine analoges (tNRTI) in particular [22–25]. The tNRTIs stavudine and more recently zidovudine gradually decrease SAT mass. Typically, SAT initially increases during the first 4–8 months of therapy, but thereafter, a 19% decrease in limb fat per year has been described with stavudine and didanosine containing regimens as compared to a decrease of 1.7% per year with zidovudine and lamivudine [26]. Further evidence demonstrating the deleterious effects of tNRTIs on SAT arises from the so called “switch” studies. Replacing tNRTI by abacavir or tenofovir has in several studies lead to an increase of 300–500 g of limb fat during the first 6–12 months after the switch [27–29]. Although most data indicate a major role of tNRTIs in the development of lipoatrophy, other drug classes may be involved. Irrespective of the NRTI backbone, nelfinavir (a protease inhibitor) was associated with more severe fat loss than efavirenz (nonnucleoside reverse transcriptase inhibitor) [30], whereas in another study efavirenz caused more fat loss than lopinavir/ritonavir (protease inhibitor) [31]. The less significant role of protease inhibitors (PIs) for lipoatrophy is demonstrated in the switch studies. In contrast to the beneficial effects of switching away from tNRTIs, the effects of switching away from PIs have been disappointing regarding lipoatrophy [32].

The potential pathophysiological mechanisms leading to lipoatrophy include NRTI-induced inhibition of mitochondrial (mt) DNA polymerase gamma through several different mechanisms [33, 34]. This inhibition would lead to decreased mtDNA content which consequently would result in depletion of proteins encoded by mtDNA and dysfunctional mitochondria. Additionally, also genes encoded by nuclear DNA are affected by NRTIs, and these drugs promote apoptosis in adipocyte cell modes in vitro [35].

In keeping with these in vitro data, human studies have shown decreased mtDNA content in adipose tissue of patients with HAART-associated lipodystrophy when compared to HIV-negative subjects, HIV-infected patients not taking HAART, or to HAART-treated patients without lipodystrophy [36–39]. However, studies in healthy subjects have shown that a 2-week exposure to NRTIs leads to a decrease in mtRNA and alters expression of several nuclear genes without a significant change in the mtDNA content [40]. Various studies have shown multiple alterations in gene expression in lipoatrophic adipose tissue such as decreased expression of several transcription factors (PPAR*γ*, SREBP-1c (sterol regulatory element-binding protein), PPAR*δ*, C/EBP*α*, and *β* (CCAAT/enhancer-binding protein)) [41, 42]. Alterations have also been described in the expression of several genes involved in lipogenesis, fatty acid, and glucose metabolism, for example, the expressions of acyl coenzyme A synthase, lipoprotein lipase, and glucose transport protein 4 are decreased in patients with HAART-associated lipodystrophy [42]. Several markers of inflammation such as interleukin 6 (IL-6), tumor necrosis factor alpha (TNF*α*), CD45, and CD68 have been shown to be increased in lipoatrophic adipose tissue [41–44]. Of the adipokines, the expression of adiponectin in adipose tissue and its circulating concentration have been shown to be decreased in several studies [44–47], whereas serum concentrations of leptin have been either decreased [48, 49], unchanged [46, 50, 51], or increased [52] in lipoatrophic patients. In addition to these findings implying severe adipose tissue dysfunction, increased rate of apoptosis has also been described in the SAT of these patients [43, 53].

Although multiple alterations have been described in lipoatrophic adipose tissue, the role and sequence of each critical abnormality eventually leading to loss of SAT still remain elusive. It also remains unknown which critical abnormalities should be counteracted in order to reverse HAART-associated abnormalities in adipose tissue of these patients, and whether thiazolidinediones would have this potential.

## 4. Thiazolidinediones for HAART-Induced Metabolic Adverse Effects

Thiazolidinediones have been used in 14 clinical trials in HIV-infected, HAART-treated patients [54–67]. The basic characteristics of these trials are given in Table 1. In total, 281 patients have used rosiglitazone, 82 patients pioglitazone, and 6 patients troglitazone in these trials. Four of these trials were open label-uncontrolled studies [54, 59, 64, 67], 6 were randomized placebo-controlled studies [55–57, 61, 62, 66], and 4 had a comparison arm with another active agent (metformin or fenofibrate) [58, 60, 63, 65]. Followup times varied from 6 weeks to 12 months.

The presence of lipoatrophy without reference to insulin resistance was the inclusion criteria in five studies [55, 56, 58, 64, 66], one additional study required the presence of at least one feature of lipodystrophy (but not necessarily lipoatrophy) without reference to insulin resistance [62]. Insulin resistance (defined by fasting insulin concentration, oral glucose tolerance test, or clamp studies) without reference to lipodystrophy was the inclusion criteria in three studies [54, 63, 65]. One study required the presence of both lipoatrophy and insulin resistance [57], and another study included patients with changes in body fat (but not necessarily lipoatrophy) together with insulin resistance [60]. The troglitazone study included patients with newly diagnosed diabetes with lipodystrophy and dyslipidemia [67], and two studies did not specify any metabolic abnormalities in the inclusion criteria [59, 61]. The exclusion criteria were variable, but often included liver function tests >2-3 times upper limit of normal, serum creatinine >1-2 times upper limit of normal, haemoglobin <90–95 g/L, serum triglycerides >10–15 mmol/L, presence of heart failure, and pregnancy.

### 4.1. Effects on Body Composition

Body composition data from the eight studies which included a control arm and an objective measurement of subcutaneous fat (dual-energy X-ray absorptiometry [DEXA], computed tomography [CT], magnetic resonance imaging [MRI]) are included in Table 2. No significant changes in the amount of subcutaneous fat could be detected in four studies: two of these studies used a single method to measure adipose tissue volume (one with MRI [55], one with DEXA [62]), and the other two studies measured fat volume using both DEXA and CT [56, 65]. In contrast to these four studies, three other studies reported statistically significant increases in SAT in patients taking either rosiglitazone [57, 60] or pioglitazone [66] as compared to placebo. SAT was quantified by both DEXA and CT scan in all these three studies, but none of the studies could confirm the statistically significant increase in SAT versus placebo by the second method in the same study. The absolute changes in SAT in the thiazolidinedione arm were reported in two studies: an increase of 50 g in leg fat mass after 12-week treatment with rosiglitazone [57], and 380 g increase in limb fat mass in the pioglitazone arm after 48 weeks of therapy [66]. In the study by van Wijk et al. rosiglitazone was compared to metformin without a placebo arm [58]. In this study, there was a statistically significant increase in SAT measured by CT scan in the rosiglitazone arm versus baseline, and also relative to metformin. DEXA scanning was not performed in this study.

One study found a statistically almost significant increase in visceral adipose tissue (VAT) in the rosiglitazone group when compared to placebo [60], while there were no significant changes in VAT between the glitazone and placebo arms in the other studies. None of the placebo-controlled trials reported significant changes in BMI either within the glitazone arm or between the placebo and glitazone group, although the difference approached statistical significance in the pioglitazone trial by Slama et al. [66]. In the study comparing rosiglitazone and metformin, there was a significant increase in body mass index (BMI) within the rosiglitazone arm from baseline, and also the change between the rosiglitazone and metformin arm was statistically significant [58]. The studies finding no significant increase in SAT reported changes in body weight ranging from a loss of 3.0 kg to the gain of 3.8 kg in the thiazolidine arm [55, 56, 62, 65]. In contrast to variable effects seen on body weight with glitazones, all three studies having a metformin arm [58, 60, 63] reported a decrease in body weight from 1.2 to 2.2 kg in patients using metformin.

The effects of thiazolidinediones on body composition in HAART-treated patients contrast data from HIV-negative diabetic patients. In these patients, thiazolidinediones have increased body weight constantly by 2–4 kg after 16–26 weeks of therapy [12]. This increase has been attributed mainly to an increase in the fat mass and in some patients to edema [12]. The increase in total fat mass has been in the order of 1.5–4 kg after 3-4 months of rosiglitazone therapy [9, 68, 69], and it consists almost exclusively of the increase in the subcutaneous fat depot [7, 8, 69, 70]. Similar effects on body fat have also been described in nondiabetic patients treated with pioglitazone for insulin resistance [71] or nonalcoholic steatohepatitis [72].

Table 3 lists some confounding factors that possibly could explain the conflicting results of thiazolidinediones on SAT in different trials with HIV-infected, HAART-treated patients. The drug dose, study duration, inclusion criteria and baseline BMI appeared to be similar between studies showing a statistically significant increase versus those not showing a change in SAT. The prevalence of concomitant use of stavudine, the NRTI most strongly associated with fat loss, may explain some of the discrepancies, since the studies reporting an increase in SAT were those with least frequent use of stavudine [57, 58, 66]. Also in the study by Carr et al., after 24 weeks of treatment with rosiglitazone, there was a statistically almost significant increase in SAT in those patients not taking stavudine or zidovudine when compared to the placebo group (+480 g versus 190 g, *P* = .06), but this difference was not maintained at week 48 [56]. Similarly, in the study by Slama et al., patients not taking stavudine at baseline had a mean increase of limb fat mass of 450 g in the pioglitazone group versus 40 g increase in the placebo group (*P* = .013) [66]. Based on these data one can hypothesize that thiazolidinediones may have a fat-inducing effect in lipoatrophic SAT, but the ongoing stavudine (and zidovudine) treatment may negate this beneficial effect.

In addition to quantifying adipose tissue compartments, liver fat content was measured in one study [55]. Liver fat decreased with rosiglitazone and increased with placebo (−2.1% versus +2.1% in the rosiglitazone versus placebo, *P* < .05) [55]. Serum alanine aminotransferase (ALT) concentrations were reported in 5 rosiglitazone studies [55–58, 60]. In three of these studies, there was a statistically significant decrease in ALT concentration in the rosiglitazone arm compared either to the baseline value or to the placebo arm possibly suggesting a decrease in liver fat content [55, 56, 58].

Outside these trials, a single case report describes development of several dozen lipomas in a patient with HAART-associated lipoatrophy during 3-month therapy with rosiglitazone. After rosiglitazone was discontinued, all but 5 lipomas resolved completely [73].

### 4.2. Effects on Insulin Resistance and Blood Lipids

The effects of thiazolidinediones on glycemic indeces in the ten comparative studies reporting data on glucose and insulin are shown in Table 4. In contrast to the very modest effects on SAT described above, eight [55–58, 60, 61, 63, 65] out of 10 studies showed improvements in insulin resistance in the thiazolidinedione arm when compared to the baseline value or to the placebo arm. One study reported a significant positive correlation between the change in fasting insulin concentration and the change in liver fat content [55]. Only two studies did not show significant improvements in insulin sensitivity [62, 66]. Of note, insulin resistance was an inclusion criteria in only four [57, 60, 63, 65] out of these 10 studies.

Since none of the comparative studies recruited patients with type 2 diabetes, it is difficult to compare these effects on glycemic indeces in HAART-treated patients to those in HIV-negative diabetic patients treated with glitazones. The average decrease in haemoglobin A_1c_ has been 1–1.5% in non-HIV patients with type 2 diabetes treated with glitazones [12].

The effects of thiazolidinediones on blood lipids in the nine comparative studies reporting data on cholesterol and triglycerides are shown in Table 5. Five [55–58, 60] out of seven studies with rosiglitazone reported a statistically significant increase in total cholesterol concentration in the rosiglitazone arm when compared either to the baseline value or to the comparative arm. The absolute increases in total cholesterol concentration in the rosiglitazone arms varied from 0.4 to 1.4 mmol/L. Neither of the two pioglitazone studies reported significant changes in total cholesterol concentrations [65, 66]. HDL (high-density lipoprotein) cholesterol concentration increased statistically significantly in both studies with pioglitazone (increases of 0.09 and 0.15 mmol/L) [65, 66], but decreased significantly in two out of the seven rosiglitazone studies with absolute decreases of 0.1 and 0.15 mmol/L [58, 60]. LDL (low-density lipoprotein) cholesterol was measured in five rosiglitazone studies [56–58, 60, 62]. Four of these studies reported significant increases in LDL cholesterol concentrations when compared to the baseline value (absolute increases between 0.2–0.8 mmol/L) or to the comparative arm [56–58, 60]. The increase of 1.7 mmol/L in LDL cholesterol was statistically almost significant in one of the two pioglitazone studies [65]. Statistically significant increases in triglyceride concentrations were reported in three out of seven rosiglitazone studies (versus baseline or versus the comparative arm) [55, 56, 58]. The absolute increases were between 0.5–3.0 mmol/l. There were no significant changes in triglyceride concentrations in the two pioglitazone studies. A proatherogenic effect of rosiglitazone on blood lipids was further described by Hadigan et al. [74]. Rosiglitazone treatment increased significantly the concentration of small dense LDL cholesterol, and decreased the concentration of large HDL cholesterol and also of HDL particle size [74]. In contrast, pioglitazone treatment was associated with an increase in the LDL particle size (from 19.9 at baseline to 20.6 nm at 12 months, *P* = .06) [65].

The results of these studies with HAART-treated patients suggest pioglitazone to have a more favorable lipid profile than rosiglitazone as has been observed in patients with type 2 diabetes [12]. A striking difference in HAART-treated patients relative to HIV-negative diabetic patients was the significant increase in triglyceride concentration by 1.5 to 2.3 mmol/L in the rosiglitazone arm in some studies [55, 56]. One may hypothesize that the increases in serum triglycerides were possibly aggravated by the high prevalence of stavudine use in these studies. The ongoing stavudine treatment may have prevented the storage of circulating lipids within the adipocytes. In the study by Sutinen et al., one patient had to discontinue rosiglitazone treatment due to serum triglyceride increase up to 32.5 mmol/L [55] and another patient using rosiglitazone in the study by Cavalcanti et al. discontinued due to abnormal lipid values [62]. Carr et al. reported grade 3-4 increases in triglyceride concentrations in 57% of the participants in the rosiglitazone arm compared to 36% in the placebo arm [56]. The large study by Cavalcanti et al. did not report any deleterious effects on blood lipid concentrations by rosiglitazone, but in the same study 15% of patients in the rosiglitazone arm started lipid-lowering therapy during the study compared to 5% in the placebo arm [62]. In addition to these prospective clinical trials, a small retrospective study reported effects of fenofibrate alone versus fenofibrate in combination with rosiglitazone in HIV-infected patients [75]. When fenofibrate was given alone, triglyceride concentrations decreased by 27% and HDL cholesterol increased by 19%. In contrast, when fenofibrate was combined with rosiglitazone, triglycerides increased by 48% and HDL cholesterol decreased by 33% [75].

Three of the rosiglitazone studies [57, 60, 76] also reported the effects of rosiglitazone on free fatty acid (FFA) concentrations. In two of these studies, a statistically significant decrease in FFA concentration was found in the rosiglitazone arm when compared to baseline value or to placebo [57, 76].

Serum adiponectin concentration was measured in six rosiglitazone studies [56–58, 60, 61, 76]. All these studies showed a statistically significant increase of 0.8–4.1 *μ*g/ml in the rosiglitazone arms. One study found an inverse correlation between the change in adiponectin concentration and the change in fasting insulin concentration and liver fat content [76].

Additional findings from these clinical trials with HAART-treated patients include either a decline [77] or no change [55, 78] in leptin concentration with rosiglitazone. Circulating concentrations of inflammatory markers (TNF*α*, C-reactive protein, or IL-6) did not change in any study reporting these measurements [78–80]. Plasma concentrations of PAI-1 (plasminogen activator inhibitor-1) and tPA (tissue plasminogen activator) were reported to either decline [79] or remain unchanged [78] with rosiglitazone therapy. Plasma resistin concentration decreased with rosiglitazone in one study [78]. Rosiglitazone decreased systolic blood pressure, but had a nonsignificant effect on flow-mediated arterial dilatation compared to placebo in one study [77].

### 4.3. Thiazolidinedione-Induced Gene Expression in SAT in HAART-Treated Patients

Two studies have evaluated the effects of rosiglitazone on gene expression in SAT in HAART-treated patients [76, 81]. Sutinen et al. reported statistically significant increases in the expression of adiponectin and PPAR*γ* coactivator 1 (PGC-1), and a decrease in IL-6 expression with rosiglitazone treatment [76]. In addition, there was a significant increase in PPAR*γ* expression in the rosiglitazone arm relative to the placebo arm [76]. Mallon et al. studied gene expression in SAT two and 48 weeks after rosiglitazone or placebo treatment, and compared patients taking tNRTIs at baseline to those without tNRTI treatment [81]. After two weeks, only those randomized to rosiglitazone in the no-tNRTI group experienced a significant rise in PPAR*γ* and PGC-1 expression. At 48 weeks, PPAR*γ* expression was increased in the no-tNRTI groups when compared to tNRTI groups, but there was no difference between the rosiglitazone and placebo arms [81].

The increase in the expression of adiponectin, PPAR*γ* (albeit limited), and that of PGC-1 are consistent with data from type 2 diabetic patients treated with glitazones [82, 83]. There are, however, also some differences in these two patient populations. In non-HIV patients, the expression of lipoprotein lipase (LPL) [82, 84] and adipocyte fatty acid binding protein (aP2) [83] increased with glitazone treatment. The expression of these genes remained unchanged in HAART-treated patients [76].

Taken together, it seems plausible that thiazolidinedione treatment had a functional effect in lipoatrophic SAT by increasing the production of adiponectin and decreasing IL-6 expression. Both of these changes may have been involved in the improvement of whole body insulin sensitivity. The correlation between the change in adiponectin concentration and the change in liver fat content implies a possibility for adiponectin to have mediated this beneficial effect on liver fat [76]. These functional changes in adipose tissue occurred despite the lack of a significant increase in fat mass. One may also hypothesize that the blunted increase in PPAR*γ* expression, especially in patients receiving tNRTI therapy, as well as the lack of an increase in LPL and aP2 expression may all have contributed to the nonsignificant effect on SAT mass and to high-serum triglyceride concentration in these patients.

### 4.4. Safety

Since in vitro and animal models, as well as clinical studies clearly indicate that thiazolidinediones correct endothelial dysfunction, suppress chronic inflammatory processes, reduce fatty streak formation, and enhance plaque stabilization and regression [85], one would expect favorable effects on cardiovascular endpoints also in human studies. In contrast, the meta analysis by Nissen and Wolski [86] demonstrated that rosiglitazone as compared to the control group significantly increased (and not decreased) the odds ratio for myocardial infarction (OR 1.43; 95% confidence interval [CI], 1.03 to 1.98; *P* = .03), and the odds ratio for death from cardiovascular causes (OR 1.64; 95% CI, 0.98 to 2.74; *P* = .06). A more recent meta analysis confirmed the increased risk of myocardial infarction (RR 1.42; 95% CI, 1.06 to 1.91; *P* = .02) and heart failure (RR 2.09; 95% CI, 1.52 to 2.88; *P* < .001) with rosiglitazone, but found no significant increase in the risk of cardiovascular mortality (RR 0.90; 95% CI, 0.63 to 1.26; *P* = .53) [87]. In contrast to findings with rosiglitazone, a meta-analysis of pioglitazone found a decreased hazard ratio for death, myocardial infarction, or stroke in patients receiving pioglitazone when compared to those receiving control therapy (HR 0.82; 95% CI, 0.72 to 0.94; *P* = .005) [88]. However, the hazard ratio for serious heart failure was increased in patients receiving pioglitazone versus the control patients (HR, 1.41; 95% CI, 1.14 to 1.76; *P* = .002) [88]. Whether these deleterious effects of glitazones on cardiovascular morbidity would be diminished in patients receiving HAART, since patients are usually younger, fewer have diabetes, and so forth, or enhanced since HAART by itself increases risk for myocardial infarction [4], remains to be studied. None of the studies with HAART-treated patients using glitazones so far have reported any significant cardiovascular events.

In general, both rosiglitazone and pioglitazone were well tolerated in all trials with HAART-treated patients. However, the total number of HAART-treated patients taking rosiglitazone and pioglitazone was only 281 and 82, respectively, and none of the studies had followup beyond one year. Furthermore, it is important to keep in mind that due to exclusion criteria of these trials there are basically no data on glitazones in HIV-infected patients with significantly increased liver function tests, high creatinine or triglyceride concentrations, or low hemoglobin at baseline; all these laboratory abnormalities are relatively common in HAART-treated patients.

Regarding the known adverse effect profile of thiazolidinediones in non-HIV infected patients, it was reassuring that no cases of clinically significant oedema, heart failure or other cardiovascular events were reported. A decrease in haemoglobin concentration is another known side effect with all glitazones, which is not explained by hemodilution but possibly caused by mild suppressive effect on bone marrow [89, 90]. This might be of significance in HAART-treated patients, since both HIV per se and antiretroviral agents may cause bone marrow suppression [91]. A statistically, but not clinically significant decrease in haemoglobin concentration in the rosiglitazone arm was reported in one study, possibly also reflecting good adherence to study medication [55]. A single case with a decrease of haemoglobin concentration to less than 110 g/L was reported by Hadigan et al. [57]. Given the concerns for severe liver toxicity induced by troglitazone [92], liver function tests were carefully monitored in these patients receiving polypharmacy. A single participant in a pioglitazone trial discontinued the study due to an increase in liver function tests >3 times upper limit of normal [65]. In contrast, three studies observed significant decreases in ALT concentrations either within the rosiglitazone arm or when compared to placebo [55, 56, 58]. Adverse effects on blood lipids were already discussed earlier; two patients had to discontinue rosiglitazone due to abnormal lipid values [55, 62].

Harmful effects of thiazolidinediones on bone metabolism have recently been discussed in patients with type 2 diabetes. A recent analysis of the data from five glitazone studies suggests that treatment with thiazolidinediones, primarily rosiglitazone, contributes to bone loss [93]. This effect appears to be most prominent in postmenopausal women [93]. The effect on bone density may have special relevance in HAART-treated patients, since both HIV-infection as such and also antiretroviral therapy have been associated with decreased bone mineral density [94]. None of the studies using glitazones in HAART-treated patients have reported effects on bone density.

Potential for drug-drug interactions must always be considered, when new medications are combined with HAART. Most PIs and nonnucleoside reverse transcriptase inhibitors (NNRTIs) are not only metabolized by CYP450 3A4 but are also either inhibitors (PIs, ritonavir in particular) or inducers (NNRTIs) of the same enzyme and to lesser extent of other isoforms of CYP450 [95]. Both rosiglitazone and pioglitazone are predominantly metabolized by CYP450 2C8 (http://www.emea.europa.eu/). Rosiglitazone is not an inducer of any tested human CYP450 isoforms, but has shown moderate inhibition of 2C8 and low inhibition of 2C9 (http://www.emea.europa.eu/). There is no in vitro evidence that pioglitazone would either inhibit or induce any of the human CYP450 isoforms (http://www.emea.europa.eu/). Interaction studies have not shown clinically significant interactions with rosiglitazone and substrates for CYP450 3A4. These interactions are not expected with pioglitazone either (http://www.emea.europa.eu/).

There are currently very limited pharmacological data on the concomitant use of thiazolidinediones and antiretroviral drugs. Data from a limited number of patients by Oette et al. suggest that rosiglitazone could be safely administered together with either lopinavir or efavirenz [96]. Rosiglitazone, however, seemed to decrease nevirapine concentrations and the authors recommend to monitor nevirapine serum concentrations if these drugs are used concomitantly [96]. Serum PI concentrations were measured in one study with rosiglitazone [55] and both PI and NNRTI concentrations were measured in one pioglitazone study [66]. Neither study observed any significant change in the serum concentrations of these antiretroviral drugs during the study period. None of the studies reported any statistically significant changes in either HIV viral load or CD4 count within the glitazone arm or between different study arms. Nevertheless, six patients in the pioglitazone arm versus two in the placebo arm (*P* = .1) experienced viral breakthrough ( >400 copies/ml) during a pioglitazone trial [66]. It is not reported if these patients were possibly taking nevirapine-based HAART (potential interaction between nevirapine and rosiglitazone, see above). Interestingly, in vitro both PPAR*γ* (rosiglitazone, ciglitazone, troglitazone) and PPAR*α* (fenofibrate) agonists have been shown to inhibit HIV replication [97, 98].

## 5. Conclusions Regarding the Role of Thiazolidinediones in the Treatment of HAART-Associated Metabolic Complications

The available evidence does not support the use of thiazolidinediones for HAART-associated lipoatrophy although they may have beneficial effects in subgroups of patients such as those that do not receive concomitant tNRTI therapy. The lack of effect may at least partially be explained by the decreased expression of PPAR*γ* which has been demonstrated not only in SAT of lipoatrophic HAART-treated patients [41, 42], but also in SAT of healthy volunteers after only a 2-week exposure to NRTIs including a thymidine analog [40]. However, if a thiazolidinedione is used for lipoatrophy, pioglitazone should perhaps be preferred because of its more favorable effects on serum lipids. Currently, the best treatment option for HAART-associated lipoatrophy is to replace tNRTIs by abacavir or tenofovir [27–29] or possibly to avoid use of NRTI altogether [99]. However, if this causal treatment of lipoatrophy is not feasible due to HIV resistance profile or intolerance to other antiretroviral agents, one may consider using uridine, which showed a significant increase in SAT in a small randomized, placebo-controlled trial in lipoatrophic patients continuing tNRTI-based HAART [100]. Pravastatin has also been shown to increase SAT in HAART-treated patients in a single small trial [101]. However, patients in this trial were not recruited for lipoatrophy but hyperlipidemia and hence the effect of pravastatin in severely lipoatrophic patients remains to be studied. Finally and most importantly, one should aim to prevent lipoatrophy altogether by avoiding the use of tNRTIs in the primary combinations of HAART as has recently been suggested [102].

Glitazones, pioglitazone in particular, could however, be used for the treatment of HAART-associated diabetes, especially in patients with reduced amount of SAT. The consistent data on the beneficial effects on insulin sensitivity in HAART-treated patients support this, albeit none of these studies recruited diabetic patients. The direct evidence of the benefits in diabetic HAART-treated patients is therefore still lacking. When using glitazones for diabetes in HAART-treated patients, one must keep in mind the potential risk for heart failure associated with both glitazones and increased risk for myocardial infarction with rosiglitazone. Nevertheless, glitazones might be the preferred choice, since metformin has been associated with further loss of SAT in HAART-treated lipodystrophic patients [58, 103] and there are no clinical data at all on other oral antidiabetic agents in this patient population.

Finally, the potential therapeutic role of thiazolidinediones on liver-related morbidity in HAART-treated patients should be evaluated. Patients who are infected with both hepatitis C (HCV) and HIV seem to have higher liver fat content than those having HCV monoinfection [104, 105] although some controversy exists [106]. Among HIV-infected patients without chronic HCV infection, those with lipodystrophy have increased liver fat content when compared to age and BMI-matched HIV-negative subjects or HAART-treated nonlipodystrophic patients [48]. It, therefore, appears that both prevalent coinfection with HCV and HAART-induced metabolic complications put HIV-infected patients at increased risk for liver steatosis.

In HIV-negative patients with nonalcohol hepatic steatosis, 6-month treatment with pioglitazone decreased liver fat content, increased hepatic insulin sensitivity, and improved histologic findings with regards to liver steatosis, ballooning necrosis and inflammation [107]. In the study by Sutinen et al., treatment with rosiglitazone decreased significantly liver fat content when compared to placebo in HAART-treated patients, although patients were not recruited for increased liver fat but for the presence of lipoatrophy [55]. Two additional trials reported significant decreases in liver function tests in the glitazone arms [56, 58]. Although promising, it remains unknown whether glitazones would also improve inflammatory changes in the liver in these patients, since no biopsy data are so far available.

## Figures and Tables

**Table 1:** The basic characteristics of the studies with thiazolidinediones in HIV-infected, HAART-treated patients. HAART = highly active antiretroviral therapy, IR = insulin resistance, LA = lipoatrophy, OGTT = oral glucose tolerance test, LD = lipodystrophy.

Number of subjects	Study design	Study groups	Duration	Inclusion criteria	Reference
Rosiglitazone studies					

8	Open label, uncontrolled	Rosiglitazone 8 mg/d	6–12 weeks	IR (defined by clamp)	Gelato et al. [54]

30	Randomized, double blind	Rosiglitazone 8 mg/d, or placebo	24 weeks	LA (clinical definition)	Sutinen et al. [55]

108	Randomized, double blind	Rosiglitazone 8 mg/d, or placebo	48 weeks	LA (limb fat% <20%, or limb fat% at least 10% less than truncal fat%)	Carr et al. [56]

28	Randomized, double blind	Rosiglitazone 4 mg/d, or placebo	3 months	LA (clinical definition) and IR (fasting insulin >15 *μ*IU/ml, or 2 h insulin [OGTT] >75 *μ*IU/ml)	Hadigan et al. [57]

39	Randomized, open label	Rosiglitazone 8 mg/d, or metformin 2 g/d	26 weeks	LA (clinical definition)	van Wijk et al. [58]

20	Open label, uncontrolled	Rosiglitazone 4 mg/d	24 weeks	No LD or IR requirements	Feldt et al. [59]

105	Randomized, double blind	Rosiglitazone 4 mg/d, or metformin 2 g/d, or rosiglitazone + metformin, or placebo	16 weeks	IR (fasting insulin >15 *μ*IU/ml, or 2 h insulin [OGTT] >75 *μ*IU/ml, or 2 h glucose [OGTT] >7.7 mmol/L and fasting insulin >10 *μ*IU/ml) and self-reported changes in body fat (including increased waist-to-hip ratio or waist circumference)	Mulligan et al. [60]

37	Randomized, double blind	Rosiglitazone 8 mg/d, or placebo	6 months	Body mass index 19–24 kg/m^2^, no requirements on LD or IR	Haider et al. [61]

96	Randomized, double blind	Rosiglitazone 4 mg/d, or placebo	24 weeks	LD (clinical definition)	Cavalcanti et al. [62]

90	Randomized, open label	Rosiglitazone 4 mg/d, or metformin 1 g/d, or no treatment	48 weeks	IR (impaired fasting glucose or impaired glucose tolerance [OGTT], with fasting insulin >20 *μ*IU/ml)	Silič et al. [63]

Pioglitazone studies					

11	Open label, uncontrolled	Pioglitazone 45 mg/d	6 months	LA (clinical definition)	Calmy et al. [64]

14	Randomized, double blind (2 × 2 factorial)	Pioglitazone 30–45 mg/d, or fenofibrate 200 mg/d, or pioglitazone + fenofibrate, or placebo	12 months	IR (impaired glucose tolerance [OGTT], or diabetes, or fasting insulin >20 *μ*IU/ml) and dyslipidemia	Gavrila et al. [65]

130	Randomized, double blind	Pioglitazone 30 mg/d, or placebo	48 weeks	LA (clinical definition)	Slama et al. [66]

Troglitazone study					

6	Open label, uncontrolled	Troglitazone 400 mg/d	3 months	LD and newly diagnosed diabetes	Walli et al. [67]

**Table 2:** Body composition data from thiazolidinedione studies which included a control arm and an objective measurement of body composition in HIV-infected, HAART-treated patients. HAART = highly active antiretroviral therapy, SAT = subcutaneous adipose tissue, NS = nonsignificant, MRI = magnetic resonance imaging, DEXA = dual-energy X-ray absorptiometry, CT = computed tomography, s.c. = subcutaneous, ND = not done, CI = confidence interval.

	*N*	Drug	Duration	Subcutaneous adipose tissue	Visceral adipose tissue	Body mass index (kg/m^2^)	Reference
No change in SAT	30	Rosi versus placebo	24 weeks	MRI: NS	MRI: NS	NS	Sutinen et al. [55]

	108	Rosi versus placebo	48 weeks	DEXA limb fat: NS CT thigh: NS CT s.c. abdomen: NS	CT: NS	NS	Carr et al. [56]

	14	Pio versus feno versus pio + feno versus placebo	12 months	DEXA upper limb fat: NS DEXA lower limb fat: NS CT s.c. abdomen: NS	CT: NS	NS	Gavrila et al. [65]

	96	Rosi versus placebo	24 weeks	DEXA limb fat: NS DEXA arm fat: NS DEXA leg fat: NS	ND	NS	Cavalcanti et al. [62]

Increase in SAT	28	Rosi versus placebo	3 months	CT thigh (cm^2^): rosi: + 2.3 versus pla −0.9, Δ rosi versus pla, *P* = .002 CT s.c. abdomen: NS DEXA leg: NS (rosi +50 g versus pla −80 g, Δ rosi versus pla *P* = .08)	CT: NS	NS	Hadigan et al. [57]

	105	Rosi versus metformin versus rosi + met versus placebo	16 weeks	DEXA leg fat (%): rosi: +4.8,*NS; met: −3.6,*NS; rosi + met: −0.5,*NS; pla: −8.3%,*NS Δ rosi vs pla *P* = .03, other groups versus pla, NS DEXA arm fat: NS DEXA limb fat: NS CT s.c. abdomen: NS	CT (%): rosi: 0.0,*NS; met: −0.6,*NS; rosi + met: −7.9,*NS; pla: −7.2,*NS Δ rosi versus pla, *P* = .08, other groups versus pla, NS	Body mass index ND, body weight (kg): rosi: 0.0,*NS; met: −2.0,*P < .001; rosi + met: −1.5,*P < .01; pla: −0.05,*NS Δ met versus pla, *P* = .03; Δ met + rosi versus pla, *P* = .06	Mulligan et al. [60]

	130	Pio versus placebo	48 weeks	DEXA limb fat (g): pio: +380 g; pla: +50 g Δ pio versus pla *P* = .051 CT s.c. abdomen: NS	CT: NS	Pio: +0.9; pla: +0.3 Δ pio versus pla *P* = .07	Slama et al. [66]

	39	Rosi versus metformin	26 weeks	CT s.c. abdomen (cm^2^) rosi: +16,*P < .05; met: −11,*P < .05 Δ rosi versus met 27cm^2^ (95% CI, 7 to 46)	CT (cm^2^) rosi: −1,*NS; met: −25,*P < .05 Δ rosi versus met 24 cm^2^ (95% CI, 6 to 51)	Rosi: +0.4,*P < .05; met: −0.4,*P < .05 Δ rosi versus met 0.7 (95% CI, 0.5 to 1.6)	van Wijk et al. [58]

* denotes significance within the study group compared to baseline value, Δ denotes comparison of the change between respective study groups.

**Table 3:** Comparison of the baseline characteristics of the thiazolidinedione arms of the studies showing versus not showing an increase in subcutaneous fat mass in HIV-infected HAART-treated patients. HAART = highly active antiretroviral therapy, SAT = subcutaneous adipose tissue, NR = not reported.

	Drug (dose/d)	Duration	Inclusion criteria	BMI (kg/m^2^)	% taking stavudine	Reference
No change in SAT	Rosi 8 mg	24 weeks	Lipoatrophy	24	67	Sutinen et al. [55]
	Rosi 8 mg	48 weeks	Lipoatrophy	23	49	Carr et al. [56]
	Pio 30–45 mg	12 months	Insulin resistance and dyslipidemia	26	NR	Gavrila et al. [65]
	Rosi 4 mg	24 weeks	Lipodystrophy	25	NR	Cavalcanti et al. [62]

Increase in SAT	Rosi 4 mg	3 months	Lipoatrophy and insulin resistance	26	44	Hadigan et al. [57]
	Rosi 4 mg	16 weeks	Lipodystrophy and insulin resistance	Body weight 80 kg	NR	Mulligan et al. [60]
	Pio 30 mg	48 weeks	Lipoatrophy	22	25	Slama et al. [66]
	Rosi 8 mg	26 weeks	Lipoatrophy	24	21	van Wijk et al. [58]

**Table 4:** The effects of thiazolidinediones on glycemic indeces in controlled trials with HIV infected, HAART-treated patients. HAART = highly active antiretroviral therapy, HOMA = homeostasis model assessment (fasting insulin [*μ*IU/ml] × fasting glucose [mmol/L]/22.5), OGTT = oral glucose tolerance test, NS = nonsignificant, NR = not reported, AUC = area under the curve.

*N*	Drug	Duration	Insulin (*μ*IU/ml)	HOMA	OGTT	Fasting glucose (mmol/L)	Reference
30	Rosi versus placebo	24 weeks	Rosi: −3.3,*P < .05; pla: +6.7,*NS Δ rosi versus pla *P* < .05	NR	NR	NS	Sutinen et al. [55]

108	Rosi versus placebo	48 weeks	Rosi: −3.5; pla: +0.7 Δ rosi versus pla *P* = .02	Rosi: −1.0; pla: +0.04 Δ rosi versus pla *P* = .03	2 h glucose: NS 2 h insulin: rosi −13.6; pla +3.9 Δ rosi versus pla *P* = .09	NS	Carr et al. [56]

14	Pio versus feno versus pio + feno versus placebo	12 months	NS	Pio: −3.8,*P < .05; pla: −1.3,*NS	NR	NS	Gavrila et al. [65]

96	Rosi versus placebo	24 weeks	NS	NS	NS	NS	Cavalcanti et al. [62]

28	Rosi versus placebo	3 months	NS	NR	2 h glucose: rosi: −0.3; pla: +0.1 Δ rosi versus pla *P* = .06 2h insulin AUC: rosi: −2.3; pla: +1.8 Δ rosi versus pla *P* = .003	NS	Hadigan et al. [57]

105	Rosi versus metformin versus rosi + met versus placebo	16 weeks	Rosi: −4,*P = .08; met: −2,*P = .07	NR	Insulin AUC: rosi: −26,*P = .012; met: −11,*P = .06; rosi + met: −18,*P = .002 Δ rosi + met versus placebo *P* = .03; Δ rosi versus pla *P* = .07	NS	Mulligan et al. [60]

130	Pio versus placebo	48 weeks	NS	NS	NS	NS	Slama et al. [66]

37	Rosi versus placebo	6 months	NS	Rosi: −0.1,*NS; pla: +1.3,*P < .05 At 6 months: rosi versus pla *P* < .05	NR	NS	Haider et al. [61]

90	Rosi versus metformin versus No-treatment	48 weeks	Rosi: −19.3,*P < .001; met: −11.1,*P < .001; No-Tx: +0.7,*NS At 48 weeks: rosi versus No-Tx *P* < .001; met versus No-Tx *P* < .001; met versus rosi *P* < .001	Rosi: −7.3,*P < .001; met: −6.2,*P < .001; No-Tx: +0.3,*NS At 48 weeks: rosi versus No-Tx *P* < .001; met versus No-Tx *P* < .001; rosi versus met *P* < .001	NR	Rosi: −1.9,*P < .001; met: −2.2,*P < .001; No-Tx: 0.0,*NS At 48 weeks: Rosi versus No-Tx *P* < .001; met versus No-Tx *P* < .001; rosi versus met *P* = .015	Silič et al. [63]

39	Rosi versus metformin	26	NR	NR	Glucose AUC: rosi: −1.9,*P = .04; met: −1.1,*P = .05 Δ rosi versus met NS Insulin AUC: rosi: −37,*P = .01; met −33,*P = .01 Δ rosi versus met NS	NR	van Wijk et al. [58]

* denotes significance within the study group compared to baseline value, Δ denotes comparison of the change between respective study groups.

**Table 5:** The effects of thiazolidinediones on blood lipids in comparative studies with HIV infected, HAART-treated patients. HDL = high-density lipoprotein, LDL = low-density lipoprotein, NS = nonsignificant, NR = not reported, CI = confidence interval.

*N*	Drug	Duration	Total cholesterol (mmol/L)	HDL cholesterol (mmol/L)	LDL cholesterol (mmol/L)	Triglycerides (mmol/L)	Reference
30	Rosi versus placebo	24 weeks	Rosi: +1.4,*P < .01; pla: 0.0,*NS Δ rosi versus pla NS	NS	NR	NS At 12 weeks: rosi: +3.0,*P < .05; pla: NS	Sutinen et al. [55]

108	Rosi versus placebo	48 weeks	Rosi: +0.9; pla: 0.0 Δ Rosi versus pla *P* < .001	NS	Rosi: +0.8; pla: +0.4 Δ rosi versus pla *P* = .04	Rosi: +1.5; pla: +1.3 Δ rosi versus pla *P* = .04	Carr et al. [56]

14	Pio versus feno versus pio + feno versus placebo	12 months	NS	pio: +0.15,*NS; pla: −0.20,*NS Δ pio versus pla *P* = .01	Pio: +1.7,*P = .07; pla: −0.6,*NS	NS	Gavrila et al. [65]

96	Rosi versus placebo	24 weeks	NS	NS	NS	NS	Cavalcanti et al. [62]

28	Rosi versus placebo	3 months	Rosi: +0.6; pla: −0.4 Δ rosi versus pla *P* = .007	NS	Rosi: +0.4; pla: −0.4 Δ rosi versus pla *P* = .01	NS	Hadigan et al [57]

105	Rosi versus metformin versus rosi + met versus placebo	16 weeks	Rosi: +0.4,*P < .05; all other groups*NS	Rosi: −0.1,*P < .001; all other groups*NS Δ rosi versus pla *P* = .005; Δ rosi versus rosi + met *P* = .006	Rosi: +0.2,*P < .05; all other groups*NS Δ rosi versus pla *P* = .048	NS	Mulligan et al. [60]

130	Pio versus placebo	48 weeks	NS	Pio: +0.09; pla: −0.08 Δ pio versus pla *P* = .005	NS	NS	Slama et al. [66]

37	Rosi versus placebo	6 months	NS	NR	NR	NS	Haider et al. [61]

39	Rosi versus metformin	26 weeks	Rosi: +0.4,*NS; met: −0.4,*P < .05 Δ rosi versus met 0.8 (95% CI, 0.3 to 1.3)	Rosi: −0.15,*P < .05; met: +0.01,*NS Δ rosi versus met 0.16 (95% CI, −0.35 to −0.02)	Rosi: +0.2,*NS: met: −0.4,*P < .05 Δ rosi versus met 0.6 (95% CI, 0.2 to 1.1)	Rosi: +0.5,* versus <0.05; met: −0.6,*P < .05 Δ rosi versus met 1.1 (95% CI, 0.4 to 2.6)	van Wijk et al. [58]

* denotes significance within the study group compared to baseline value, Δ denotes comparison of the change between respective study groups.
